# Association of preoperative elevated lipoprotein (a) with poor survival in patients with biliary tract cancers

**DOI:** 10.1002/cam4.7331

**Published:** 2024-05-31

**Authors:** Shanshan Fan, Yihan Mao, Yang Ge, Ziwei Liang

**Affiliations:** ^1^ Department of Oncology, Beijing Chao‐Yang Hospital Capital Medical University Beijing China; ^2^ The Third Clinical School of Medicine Capital Medical University Beijing China

**Keywords:** biliary tract cancers, lipid metabolism, lipoprotein (a), prognosis, survival

## Abstract

**Background:**

Biliary tract cancers have garnered significant attention due to their highly malignant nature. The relationship between abnormal lipid metabolism and tumor occurrence and development is a research hotspot. However, its correlation with biliary tract cancers is unclear.

**Methods:**

We enrolled 78 patients with biliary tract cancers and obtained data on clinical characteristics, pathological findings, and preoperative blood lipid indices, including total cholesterol (TC), high‐density lipoprotein cholesterol (HDL‐C), low‐density lipoprotein cholesterol (LDL‐C), triglycerides (TG), and lipoprotein (a) [Lp(a)]. Receiver operating characteristic (ROC) curves were used to determine the optimal predictive cutoff values of lipid indicators among the participants. Independent risk factors were determined using Cox regression, and survival was predicted using the Kaplan–Meier method. Statistical analyses were performed using SPSS software.

**Results:**

Univariate Cox regression analysis revealed that the body mass index (BMI), tumor location, surgical margin, N stage, and abnormally increased LDL‐C, TG, and Lp(a) levels were significantly associated with poor prognosis of biliary tract cancers (*p* < 0.05). Multifactor Cox regression demonstrated that only N stage (HR = 3.393, *p* < 0.001) and abnormally increased Lp(a) levels (HR = 2.814, *p* = 0.004) were significantly associated with shorter survival. N stage and Lp(a) were identified as independent prognostic risk factors for patients with biliary tract cancers.

**Conclusion:**

This study presents Lp(a) as a novel biochemical marker that can guide clinical treatment strategies for patients with biliary tract cancers. More effective treatment options and intensive postoperative testing should be considered to prolong the survival of these patients with preoperative abnormal lipid metabolism.

## 1 INTRODUCTION

Biliary tract cancers account for approximately 3% of all gastrointestinal malignancies, and its incidence is increasing globally.^1^ Based on their anatomical structure, these cancers encompass gallbladder cancer (GC), intrahepatic cholangiocarcinoma (ICC), or extrahepatic cholangiocarcinoma (ECC).^2^ While radical surgical resection is an effective treatment for early biliary tract cancers, for patients with locally advanced or metastatic disease, clinicians may resort to chemotherapy,^3^ immunotherapy,^4^ and targeted therapy,^5^ with a modest 5‐year survival rate ranging from 5% to 15%.^6^ Serum CEA and CA199 levels are crucial in determining and monitoring the efficacy, metastasis, and recurrence of biliary tract cancers.^7^ However, novel predictors for patient survival remain elusive. Abnormal metabolism in tumor cells, a characteristic of malignant tumors,^8^ has garnered considerable research focus lately. Lipid metabolism disorders are closely related to the occurrence and development of gastrointestinal malignancies; however, the underlying mechanisms remain unclear.^9^ Active lipid metabolism reportedly enhances energy production in tumor cells, which is conducive to their survival in nutrient‐poor microenvironments. Moreover, signaling molecules produced during abnormal lipid metabolism can activate tumor‐related signaling pathways and promote the proliferation, invasion, and metastasis of tumor cells.^10^

Lp(a), akin in structure to LDL‐C, exhibits ethnic‐related differences.^11^ It has been shown to be a risk factor for cardiovascular diseases.^12^ However, its correlation with biliary tract cancers is rarely reported. Therefore, in the present study, we conducted survival analyses with an aim to examine this correlation in patients with biliary tract cancers.

## 2 METHODS

### 2.1 Study design and patients

**FIGURE 1:**
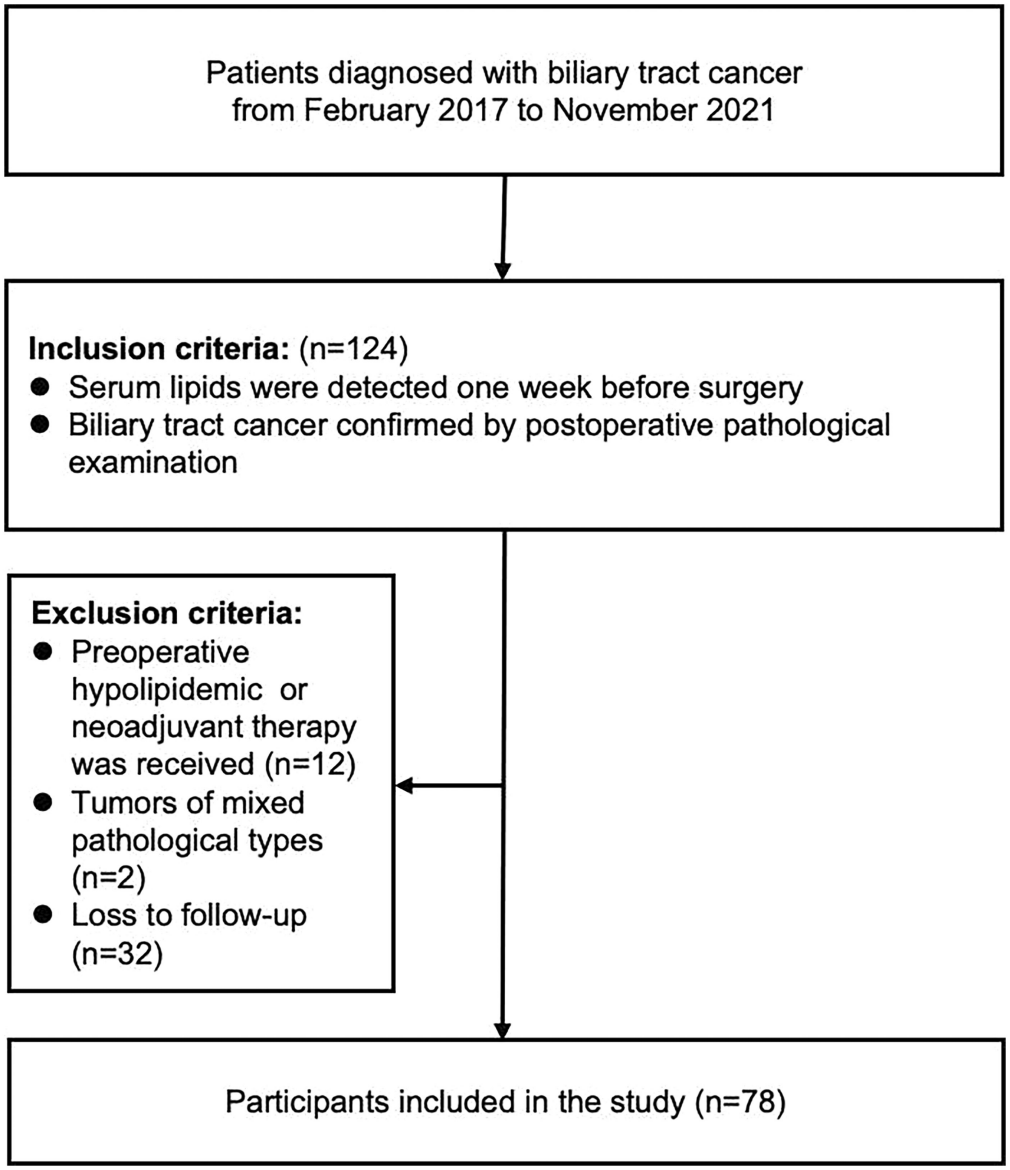
Flowchart depicting the inclusion of patients with biliary tract cancers.

In this retrospective study, we collected data on patients with pathologically diagnosed biliary tract cancers who were being treated at Beijing Chao‐Yang Hospital, Capital Medical University, between February 2017 and November 2021. We initially evaluated the eligibility of 124 patients with biliary tract cancers who underwent surgical resection. Given the complex anatomy of the biliary tract, the main surgical methods included the Whipple procedure, enlarged cholecystectomy, or laparoscopic palliative partial hepatectomy, among others. Detailed inclusion and exclusion criteria are depicted in Figure 1. Clinically relevant baseline data, personal history, and blood test results were collected. The patients' survival was tracked by monitoring the inpatient and outpatient medical records following their biliary tract cancer diagnosis at the hospital or via telephone. The last follow‐up was performed in May 2023.

### 2.2 Blood specimens and tests

Fasting blood samples (3 mL venous blood) were collected in the morning, 1 week before surgery using a tube without anticoagulant coating. The sample was centrifuged at 3540× g for 8 min, and the supernatant was used for further analyses. TC and TG levels were analyzed using an enzymatic method, while HDL‐C and LDL‐C levels were analyzed using direct methods. Finally, Lp(a) levels were analyzed using latex immunoturbidimetry (SIEMENS ADVIA2400, Germany).

### 2.3 Statistical analysis

Data analyses and image construction were performed using SPSS version 24.0 (IBM; Chicago, Illinois, USA). Continuous variables are expressed as means and standard deviations, and categorical variables are expressed as percentages (%). A receiver operating characteristic (ROC) curve was plotted based on sensitivity and specificity, to calculate the area under the curve (AUC) and maximum Youden's index. Survival was assessed using Kaplan–Meier curves, and differences in survival between the two groups were determined using the log‐rank test. Univariate and multivariate analyses were performed using Cox regression analysis, and potential independent risk factors were identified using logistic regression. Forest plots were constructed using GraphPad Prism version 10.0. Statistical significance was set at *p* < 0.05.

## 3 RESULTS

### 3.1 Baseline characteristics

Initially, 124 patients with a pathological diagnosis of biliary tract cancers were screened according to the inclusion criteria. Based on the exclusion criteria, 12 of the patients diagnosed with hyperlipidemia before surgery who had received oral lipid‐lowering drugs were excluded. Additionally, the postoperative pathology of two patients was complicated by other pathological components, and 32 patients were lost during follow‐up. Consequently, a total of 78 patients met the study criteria and were included (Table 1).

**TABLE 1:** Basic clinical characteristics of the patients with biliary tract cancers included in this study.

Variable	Total (*N* = 78) *n* (%)
Age (years)	
≥70	30 (38.5%)
<70	48 (61.5%)
Sex	
Male	42 (53.8%)
Female	36 (46.2%)
BMI (Kg/m^2^)	
Normal (18.5–24)	35 (44.9%)
Overweight (≥24)	30 (38.5%)
Obese (≥28)	9 (11.5%)
Underweight (≤18.5)	4 (5.1%)
HBP	
Yes	5 (6.4%)
No	73 (93.6%)
DM	
Yes	20 (25.6%)
No	58 (74.4%)
Smoking	
Yes	15 (19.2%)
No	63 (80.8%)
Drinking	
Yes	13 (16.7%)
No	65 (83.3%)
*Pathological characteristics*	
Tumor location	
GC	15 (19.2%)
ICC and/or ECC	63 (80.8%)
Surgical margin	
Negative	40 (51.3%)
Positive	38 (48.7%)
Perineural invasion	
Negative	21 (26.9%)
Positive	57 (73.1%)
Vascular invasion	
Negative	42 (53.8%)
Positive	36 (46.2%)
Grade	
G1 + G2	53 (67.9%)
G3 + G4	25 (32.1%)
T stage	
T1 + T2	42 (53.8%)
T3 + T4	36 (46.2%)
N stage	
N0	47 (60.3%)
N+	31 (39.7%)
M stage	
M0	72 (92.3%)
M1	6 (7.7%)
TNM stage	
I + II	42 (53.8%)
III + IV	36 (46.2%)

Abbreviations: BMI, body mass index; DM, diabetes mellitus; ECC, extrahepatic cholangiocarcinoma; GC, gallbladder cancer; HBP, high blood pressure; ICC, intrahepatic cholangiocarcinoma; Kg/m^2^, weight (kg)/height^2^ (m).

### 3.2 ROC analysis to determine optimal cutoff values for lipid indices

While previous studies defined abnormal lipid indices based on the risk of cardiovascular and cerebrovascular diseases (such as hyperlipidemia and atherosclerosis),^12^ in this study, we aimed to evaluate the correlation between blood lipid indicators and the prognosis of biliary tract cancers. Therefore, based on the biochemical blood lipid indicators and survival of patients, the optimal cutoff values of various lipid indices were confirmed using ROC curves. The optimal cutoff values, corresponding to the maximum Youden's index, were as follows: 5.325 mmol/L for TC, 1.380 mmol/L for HDL‐C, 3.660 mmol/L for LDL‐C, 2.370 mmol/L for TG, and 24.35 mg/dL for Lp(a).

### 3.3 Univariate and multivariate Cox regression analyses

**FIGURE 2:**
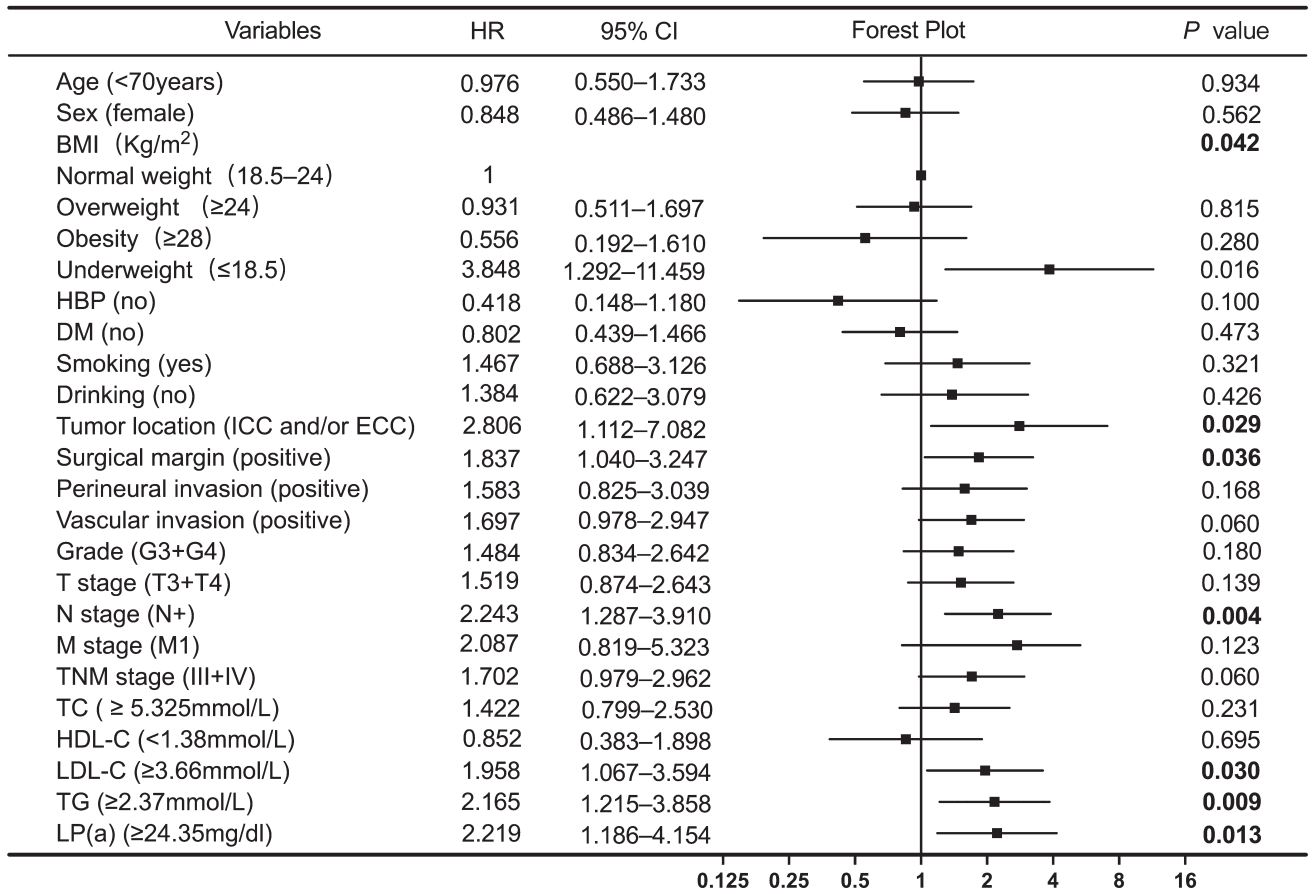
Univariate Cox regression analysis of the overall survival (OS) of patients with biliary tract cancers. †BMI, body mass index; CI, confidence interval; DM, diabetes mellitus; ECC, extrahepatic cholangiocarcinoma; HBP, high blood pressure; HDL‐C, high‐density lipoprotein cholesterol; HR, hazard ratio; ICC, intrahepatic cholangiocarcinoma; Kg/m^2^, weight (kg)/height^2^ (m); LDL‐C, low‐density lipoprotein cholesterol; Lp(a), lipoprotein (a); OS, overall survival; TC, total cholesterol; TG, triglycerides.

**FIGURE 3:**
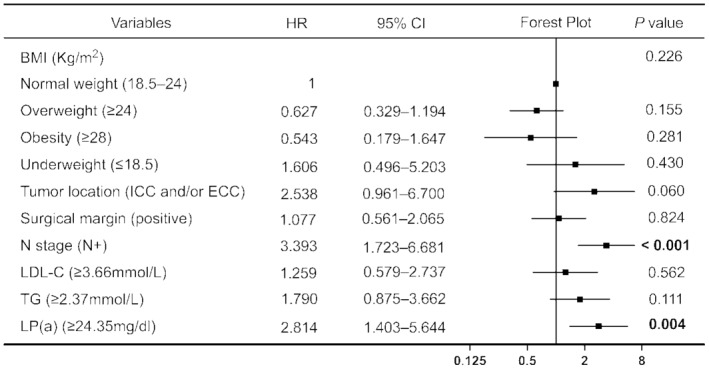
Multivariate Cox regression analysis of the patients.†BMI, body mass index; CI, confidence interval; ECC, extrahepatic cholangiocarcinoma; HR, hazard ratio; ICC, intrahepatic cholangiocarcinoma; Kg/m^2^, weight (kg)/height^2^ (m); LDL‐C, low‐density lipoprotein cholesterol; Lp(a), lipoprotein (a); TG, triglyceride.

**FIGURE 4:**
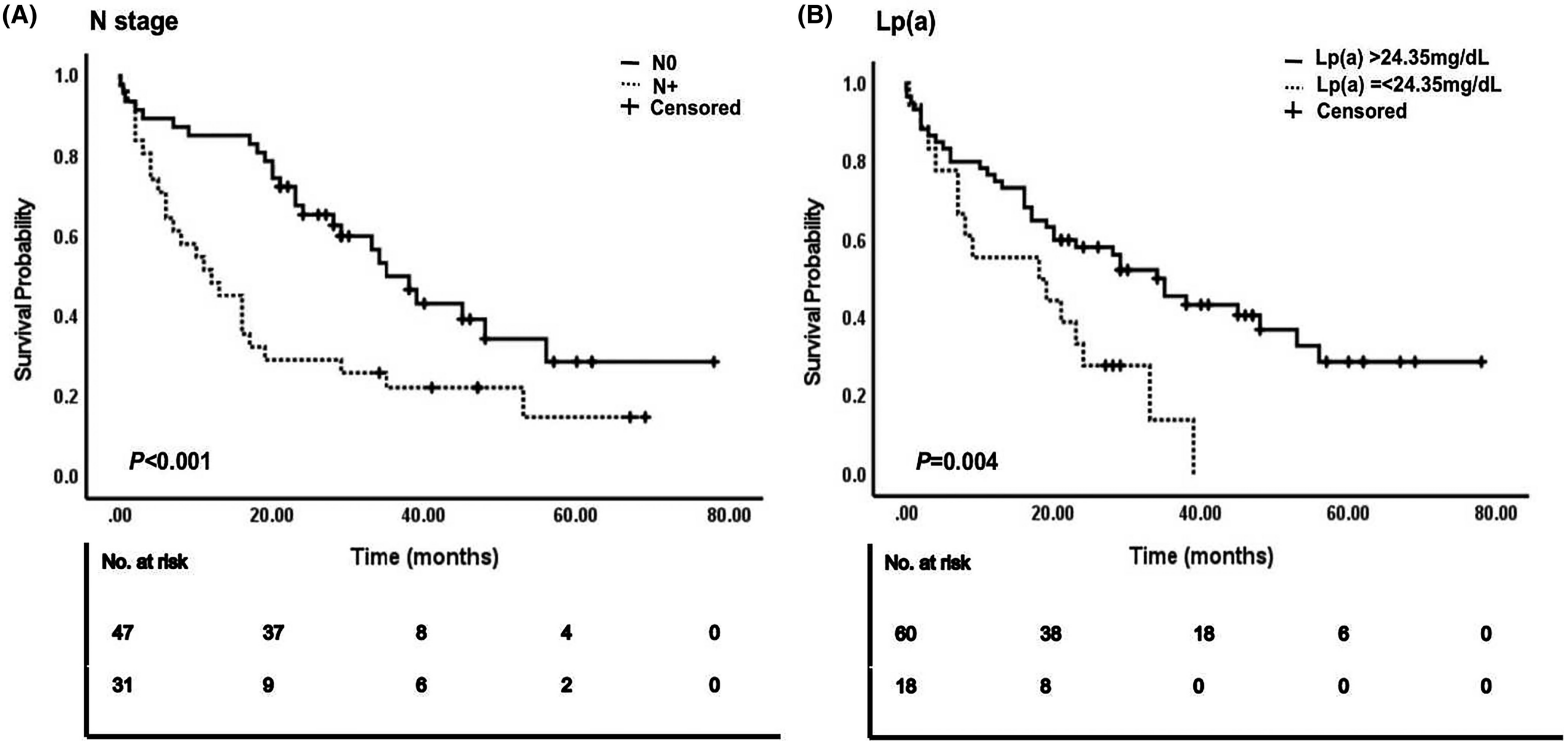
KM survival curves for independent prognostic risk factors A) N stage, (B) Lp (a).†KM, Kaplan–Meier; Lp(a), lipoprotein(a).

All variables were included in the univariate Cox regression analysis, with blood lipid indicators measured based on the optimal cutoff values determined in this study (Figure 2). The results showed that underweight patients (determined by their body mass index [BMI]) had worse prognoses than patients with normal BMI (hazard ratio [HR] = 3.848, *p* = 0.016). Notably, no significant differences were noted for overweight or obese patients. Patients with ICC and/or ECC exhibited shorter OS and poorer prognosis than those with GC; this difference was statistically significant (HR = 2.806, *p* = 0.029). The pathological results indicated that a positive tumor surgical margin (HR = 1.837, *p* = 0.036) was predictive of a poor prognosis. The OS of patients at N stage was significantly shorter than that of patients without lymph node metastasis (HR = 2.243, *p* = 0.004). Increased LDL‐C (HR = 1.958, *p* = 0.030), TG (HR = 2.165, *p* = 0.009), and Lp(a) levels (HR = 2.219, *p* = 0.013) were associated with poor prognosis. None of the other variables appeared to correlate with OS (*p* > 0.05). Variables showing significant (*p* < 0.05) correlation with OS in the univariate Cox regression analysis were included in the multivariate Cox regression analysis (Figure 3). The prognosis of patients with lymph node metastasis was poor, and the difference was statistically significant (HR = 3.393, *p* < 0.001). Additionally, patients with abnormally high Lp(a) levels (HR = 2.814, *p* = 0.004) had a poor prognosis. Figure 4 illustrates the survival curve of the independent risk factors generated using the log‐rank test and the Kaplan–Meier method.

## 4 DISCUSSION

This study aimed to investigate the correlation between blood lipid‐related markers and OS in 78 patients with biliary tract cancers. By plotting the ROC curve and calculating the optimal cutoff value based on the maximum Youden's index, we identified Lp(a) as an independent risk factor for poor prognosis in patients with biliary tract cancer. The optimal cutoff value for Lp(a) was 24.35 mg/dL, which falls below the normal range detected at our hospital (30 mg/dL). Therefore, the conventional levels used to evaluate cardiovascular disease risk may not effectively predict the survival outcomes of patients with biliary tract cancers. Thus, consideration and adjustment of the Lp(a) level as a parameter could enhance prognostic evaluation and OS prediction for these patients. In addition, we observed a significant association between the N stage and poor prognosis among patients with biliary tract cancers, which suggested it as an independent risk factor, consistent with findings from previous studies.^13^, ^14^

Elevated Lp(a) levels have been linked to more aggressive forms of prostate^15^ and colorectal cancers,^16^ while no associations have been observed with breast cancer.^17^ However, the underlying mechanisms remain unclear. Some studies have suggested that elevated Lp(a) levels may contribute to the formation of thrombi and fibrin networks, thereby promoting the adhesion, invasion, and metastasis of cancer cells.^16^ However, animal studies have shown that the protein hydrolysate of Lp(a) exhibits anti‐tumor properties both in vivo and in vitro.^18^, ^19^ This anti‐tumor effect of increased Lp(a) levels may represent a compensatory response to systemic chronic inflammation caused by malignant tumors.^15^

## 5 CONCLUSION

In conclusion, we have preliminarily identified a novel marker that can predict the clinical prognosis of patients with biliary tract cancers. Therefore, patients with biliary tract cancers with abnormally elevated Lp(a) should be more rigorously classified before surgery. Following diagnosis, timely and intensive treatment, earlier imaging examination, and more intensive imaging monitoring are necessary. Nevertheless, persistent efforts would be made to expand the sample size and validate our findings in larger cohorts of patients with biliary tract cancer. Based on this study's salient findings, further research studies should be undertaken to uncover the crucial mechanisms of lipid metabolism involved in the development of biliary tract cancers and reveal novel insights for guiding treatment strategies.

## AUTHOR CONTRIBUTIONS

**Shanshan Fan:** Data curation (equal); formal analysis (equal); investigation (lead); project administration (lead); resources (lead); software (lead); validation (lead); writing – original draft (lead); writing – review and editing (lead). **Yihan Mao:** Data curation (equal); formal analysis (equal); investigation (equal). **Yang Ge:** Project administration (equal). **Ziwei Liang:** Formal analysis (equal).

## FUNDING INFORMATION

This study received funding from the Undergraduate Scientific Research Innovation Project of Capital Medical University (project number XSKY2023255).

## CONFLICT OF INTEREST STATEMENT

There are no conflicts of interest to report.

### ETHICS STATEMENT

All participants provided informed consent. This study was approved by the Beijing Chao‐yang Hospital Ethics Committee, affiliated with Capital Medical University. All procedures followed were in accordance with the ethical standards of the responsible committee on human experimentation (institutional and national) and the Helsinki Declaration of 1964 (and its later amendments).

## Data Availability

The datasets generated during and/or analyzed during the current study are available from the corresponding author on reasonable request.
